# A Novel Computer-Assisted Approach to evaluate Multicellular Tumor Spheroid Invasion Assay

**DOI:** 10.1038/srep35099

**Published:** 2016-10-12

**Authors:** Liliana R. Cisneros Castillo, Andrei-Dumitru Oancea, Christian Stüllein, Anne Régnier-Vigouroux

**Affiliations:** 1 Institute of Zoology Johannes Gutenberg University of Mainz, Mainz, Germany; 2 CLADIAC GmbH, Heidelberg, Germany

## Abstract

Multicellular tumor spheroids (MCTSs) embedded in a matrix are re-emerging as a powerful alternative to monolayer-based cultures. The primary information gained from a three-dimensional model is the invasiveness of treatment-exposed MCTSs through the acquisition of light microscopy images. The amount and complexity of the acquired data and the bias arisen by their manual analysis are disadvantages calling for an automated, high-throughput analysis. We present a universal algorithm we developed with the scope of being robust enough to handle images of various qualities and various invasion profiles. The novelty and strength of our algorithm lie in: the introduction of a multi-step segmentation flow, where each step is optimized for each specific MCTS area (core, halo, and periphery); the quantification through the density of the two-dimensional representation of a three-dimensional object. This latter offers a fine-granular differentiation of invasive profiles, facilitating a quantification independent of cell lines and experimental setups. Progression of density from the core towards the edges influences the resulting density map thus providing a measure no longer dependent on the sole area size of MCTS, but also on its invasiveness. In sum, we propose a new method in which the concept of quantification of MCTS invasion is completely re-thought.

Tumor invasion is a complex process influenced by the interaction between tumor cells and their microenvironment. The process of invasion encompasses not only mechanical movement of the cells, but also dynamical tumor cell proliferation^1^, as well as angiogenesis and inflammation (immune cells recruitment) in the tumor bulk and invaded extra-cellular matrix (ECM)^2^. *In vivo* local invasion involves tumor cell adhesion and proteolytic remodeling of the ECM simultaneously, whereas distant metastases are observed when tumor cells mainly invade due to a high protein expression of proteases, causing a substantial spatial separation from the tumor bulk^3^. Unless the surrounding of the tumor bulk is disturbed by inhomogeneities, tumor cells will invade omni-directionally outward from the bulk. The conditions described above are obviously absent from *in vitro*, two-dimensional culture models that lack among others a proper ECM and thus relevant ECM-cell communication or even create a bias towards inflammation and proliferative behavior^4^. In order to improve the representation of the *in vivo* reality of tumor invasion, three-dimensional (3-D) *in vitro* systems have been developed to combat the shortcomings of two-dimensional models, which are prone to misinterpretation. The multicellular tumor spheroids (MCTSs) model is a common 3-D model, used to study tumor signaling mechanisms and tumor response to different platform treatments. The typical size of MCTS ranges from 200 μm to 500 μm initial diameter. Due to this size, spheroids display a high compactness of cells, which triggers the appearance of gradients of nutrients, oxygen and catabolites^5^. The lack of oxygen is a desirable characteristic of the MCTS, because a necrotic core will develop, surrounded by quiescent hypoxic and proliferating cells providing a variety of intermixed metabolic states, which are all found in *in vivo* conditions^6^. There is a variety of setups for invasion assays, in which MCTSs are deposited in an ECM-like structure, whose composition can be made up of one or several proteins (e.g. Collagen I, Collagen IV or Matrigel)^7^. Several methods have arisen to quantify the size of MCTS in invasion assays. Vinci *et al*., present the Celigo Cytometer (Nexcelom Bioscience, LCC, Massachusetts, USA)^8^, which is an approach that comes already with a device that takes images automatically of an inserted plate. Chen *et al*., present a MATLAB implemented program named MCTSSizer^9^, which can be fed with any images of MCTSs for automatic analysis, and is independent of a device. Piccinini presents a software called AnaSP, which is extracting morphological parameters based on the assumption that MCTSs are de facto spherical. A comprehensive summary about the state of the art can be found in ref. 10. All these methods share the assumption of an invasive front in the MCTS. This quantification of the area of the invasive front however brings two limitations to the execution of invasion assays. A clear invasive front is only visible in MCTSs that stay compact during invasion, which is not true for all cell types, and the invasive front is only quantifiable as long as it does not invade beyond the frame of the image.

In this work, we present our development of a high-throughput quantification method, which is independent of the composition of the used matrix, the MCTS size or cellular composition. We avoid the issue of finding and defining an invasive front by introducing the concept of quantification by density instead of by area. We propose a robust algorithm that does not segment the image as a whole, but uses different segmentation techniques for different areas of the MCTS. This way, we do not need to sacrifice the quality of segmentation on the outer area of the MCTS in order to represent the denser core area better, since the different areas are analyzed in separate steps with separate techniques. We move away from using the invaded area as a direct means of quantification since this latter excludes disperse cells in outer areas of the image from being properly represented and analysed. We furthermore introduce the concept of segmentation density, which is proportional with cell density of the image. This multi-step analysis is resistant against uneven illumination and shadows that occur especially in samples close to the edge, and is independent of the image acquisition step, which can be done with any bright-field microscope and any camera. Analysis of pictures from various invasion assays conducted with different glioma cell lines under various treatments provides the proof-of-concept of this new algorithm.

## Results and Discussion

### Description of the approach

This section describes how the micrographs of MCTSs are analyzed with the help of computer vision techniques. It further discusses the quantification of the data that provide a measure of the relative size of spheroids. This quantification goes beyond merely measuring the invaded area, because it takes into account the invasiveness of the MCTS itself. The spheroids we used as material for the micrographs were generated according to published standard protocols^8^,^11^ and the 3-dimensional nature of cell invasiveness was controlled by confocal and time lapse video microscopy (data not shown). The program developed and used in this approach was written in C#, with CImg and OpenCV as image processing libraries, which are integrated via DLLs written in C + + . Access to the program is provided through the following link: http://www.cladiac.com/downloads/public/ClADIAC_Spheroid_Analyser_Demo_v0.7.zip.

### Image Analysis

**Figure 1:**
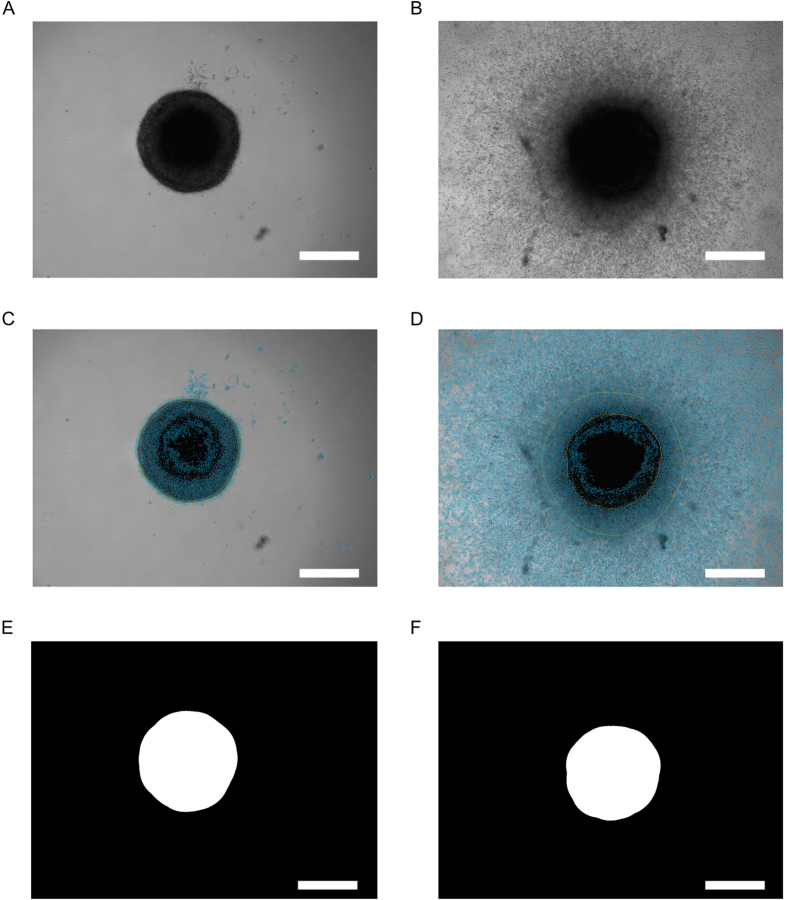
Core extraction. (**A,B**) original micrographs of the MCTSs (U87MG cells) before image processing at the beginning (**A**, day 0) and at the end (**B**, day 8) of the invasion assay. (**C,D**) representation of the calculated MCTS features in overlay after image processing. The orange line depicts the core, the green line the halo and the blue color depicts the periphery of MCTS at day 1 (**C**) and day 8 (**D**). (**E,F**) results of the core extraction on the micrographs at day 0 (**E**) and day 8 (**F**). Representative micrographs of an MCTS are shown. Scale bar = 500 μm.

**Figure 2:**
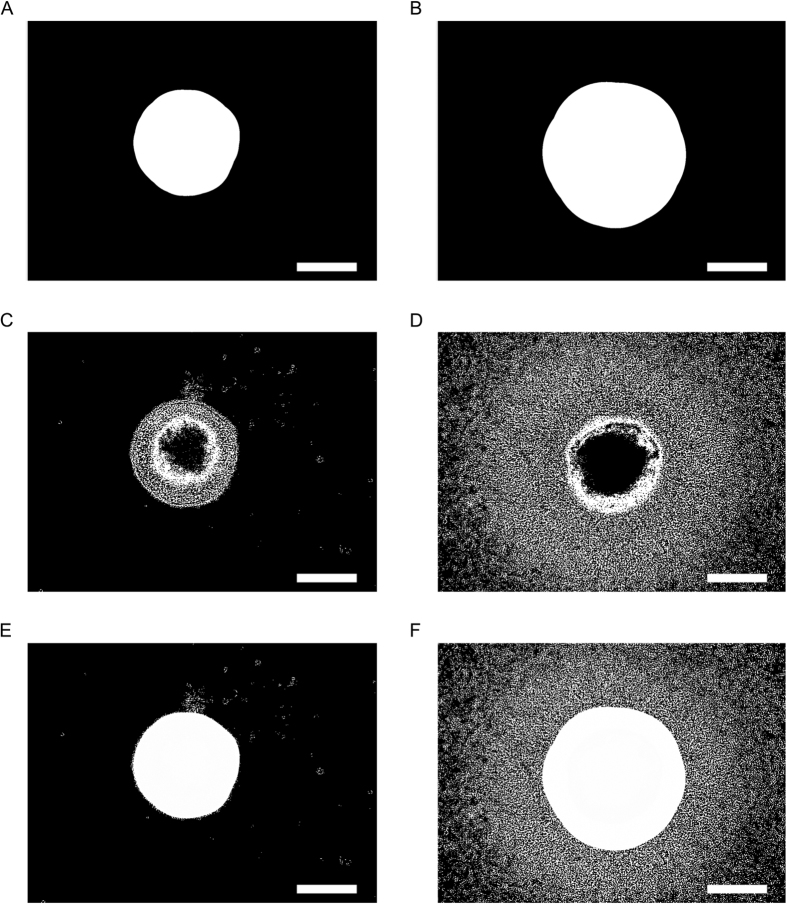
Halo extraction, periphery extraction and overlay computed images. (**A,B**) halo extraction on the micrographs of Fig. 1 at day 0 (**A**) and day 8 (**B**). (**C,D**) periphery extraction on the micrographs of Fig. 1 at day 0 (**C**) and day 8 (**D**). (**E,F**) combined overlay of the images analyzed at day 0 (**E** = overlay of 1E, 2B and 2C) and at day 8 (**F** = overlay of 1F, 2B, 2D). Scale bar = 500 μm.

MCTS micrographs have different features, which are extracted separately through different techniques to improve the quality of the measurement. Figure 1 shows two examples of images of the same MCTS analyzed at the beginning (Fig. 1A,C,E) and at the end (Fig. 1B,D,F) of the invasion assay. Figure 1A (day 0) and 1B (day 8) display the original images before applying the program. Figure 1C (day 1) and 1D (day 8) display the images after applying the program. A total of three regions are evaluated, the core (Fig. 1E,F), the halo (Fig. 2A,B) and the periphery (Fig. 2C,D), which are explained in detail below. It is important to note that these three regions are defined on the sole basis of their photographic density and not on their biological features. These latter cannot be assessed through the implementation of the software whose function is to provide an objective and observer-independent measure of the size of spheroids (and invaded areas where feasible). These regions however match with the characteristic features of spheroids, i.e. an initial and dense core or mass of cells, which with time and through division and migration, evade the core and populate the periphery. What we define by halo encompasses the core and matches with the outermost ring of the spheroid core that contains cells on their way to escape and migrate in the periphery^12^. At the starting point of the experiment, a compact core with no invaded area is correctly determined by the developed algorithm (Fig. 1E), in spite of the presence of cells or cell aggregates in the collagen. Furthermore, halo (Fig. 2A) and core (Fig. 1E) are almost identical, as expected. This is not the case at the end of the experiment: the halo of a well-developed MCTS (Fig. 2B) is here significantly bigger in size than its core (Fig. 1F) and peripheral cells have moved beyond the aperture of the camera (Fig. 2D). This image illustrates why a simple estimation of the invaded area, e.g. by cell counting or determination of outermost cells is not accurate. These latter methods are not applicable once the cells move beyond the aperture and are very inaccurate if implemented when cells are still in the boundaries of the aperture field. Defining the correct amount of cells by counting them is technically very difficult if not impossible. A light microscopy image, such as those we are using, represents a two-dimensional image that provides only a cross-section of the three-dimensional image. This cross section hides the immediate sections positioned in front of and behind it. Counting single isolated cells in the collagen matrix would be fairly easy to do and confocal microscopy would certainly improve quantitatively and qualitatively this step; however the core region is so dense that no current and affordable microscopy methods can help distinguish and count single cells. Thus, even if we would assume a uniform distribution, counting would be unreliable. Determination of the invasive front (i.e. the position of the outermost cells) is also not reliable, regardless of whether the aperture of the camera is reached or not. Indeed, we observed cases for which the outermost cells of high proliferative and low proliferative MCTSs migrated the same distance. However, even though the invasive fronts were similar, the invasion areas were different, being either densely or scarcely populated, thus reflecting different invasive behaviors. These observations and the limitations of cell counting and determination of the invasive front were the motivation for the development of our new, density-based approach. The developed approach calculates a density map from the three regions, and uses this map to draw conclusions about the MCTS. This has the further advantage that the position of the core does not have to be exactly at the same spot every time the same sample is revisited at a later time point. This enables the analysis to be performed on any given images regardless of the equipment used for obtaining them, while other software approaches may be designed to work exclusively in conjunction with a specific machine that needs to be acquired for running assays, or require manual intervention on an image-by-image level.

### Core

Figure 1C–F depicts the core segmentation of the images in Fig. 1A,B. The core is determined by Otsu’s thresholding method^13^. It is extended through plausibility checks to avoid false results by considering the shape of the found objects and selecting the most appropriate one. The image first goes through a step of image subtraction, which is able to mitigate uneven illumination of the background. In this step, the average intensity of the respective image is computed and compared to a strong Gaussian blur of the same image. The blur averages out small details of the image resulting in a brightness gradient across the original image. This blurred image is then compared to the average intensity, and each pixel of the image is either added or subtracted according to the difference in brightness between the blurred image at that pixel and the average intensity (Figure S1). As indicated by comparing the images of Fig. 1C with 1D and Fig. 1E with 1F, the core extraction gives a good core estimation for MCTSs with well-defined borders (Fig. 1C), meaning that the segmented result corresponds well with the original image. However, Otsu’s thresholding method runs into its limitations for a smoothly spread-out core (Fig. 1F), where the segmented result does not cover the dense area of the MCTS (Fig. 1D). This shows the necessity of another approach for the analysis of the area which is outside the immediate core, but still too dense to be single peripheral cells. We call this area the halo of the MCTS.

### Halo

**Figure 3:**
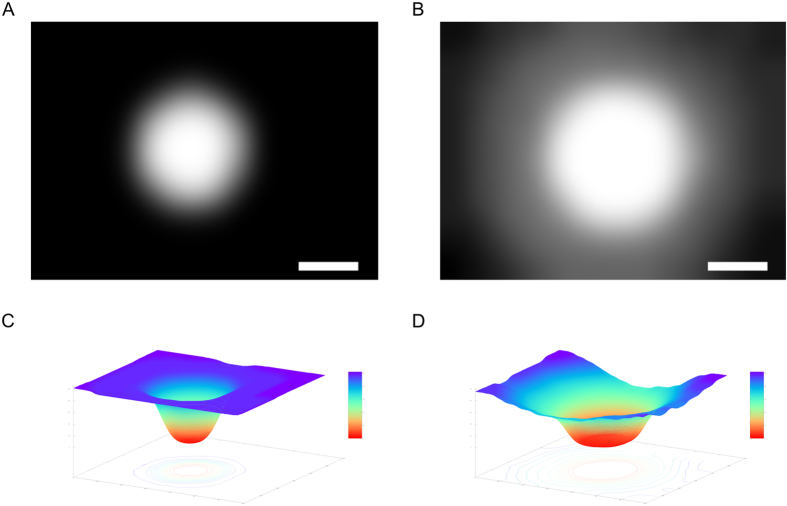
Resulting density maps and surface plots. (**A**) Density map at day 0 (**A**) and at day 8 (**B**) as well as surface plots of the determined densities at day 0 (**C**) and day 8 (**D**) are presented. Scale bar = 500 μm.

Figure 3 shows the determined halos of the sample images. The halo extraction gives the same result as the core extraction for well-defined cores (compare Figs 1E and 2A), but it extends the core into the dense surrounding region for smooth spread-out core (compare Figs 1F and 2B). Thereby it is properly representing the area size of the MCTS. The halo finding algorithm is based on a watershed algorithm, with the previously determined core as seed. The algorithm is iteratively applied to the image and it fills it step by step, starting from the core and reaching outwards, until the filled area reaches the border of the image. This is the stop criterion for the watershed algorithm; the filled area present in the step previous to the one that touches the border is taken and defined as the halo. This process is being done on the original image without prior background subtraction, because the subtraction inherently also reduces the contrast of the image. This would deteriorate the result, since the difference in intensity between the border of the segment and the border of the image is lower than in the original image. The downside is that the watershed tends to flood more into darker regions. However, the effect on the final result is negligible, since the segmented image is averaged out into a density map. Cell types that are more proliferative than invasive tend to keep clear borders during the course of assays. For these cases we also included the possibility to calculate the halo surface area in [μm^2^], provided that the measure of pixel per micron is known.

### Periphery

The result of the periphery extraction is depicted in Fig. 2C,D. The periphery is examined through a Canny edge detection^14^. The resulting binary image does not directly detect single cells. However, it is intuitive that the more single cells are in an area, the higher will be the granularity, and therefore there is an increase of determined edges. Thus, the binary image is representing the cell density around the periphery outside of the core and halo regions. For compact MCTSs, the periphery is overlapped by core and halo and does not contribute to the overall segmented image, but the impact increases as the cells spread throughout the image. This will contribute to the resulting cell density as a smooth gradient, whose intensity is proportional to the amount of peripheral cells.

### Quantification

The different regions are typically overlapping each other, which is why after extraction the segmented images are combined into an overlay (Fig. 2E,F). The combined image is split in 16 × 16 sub-images, and for each sub-image, the average cell density is calculated. It is important to note at that step that we calculate a density of the segmented image, and not of the actual image (which would imply counting the cells). A segmented image can have only one of two values for each pixel: 1 or 0 (i.e. there is a structure at the location of this pixel, or not). This is stretched to an interval of 0 to 255 (1 = 255, 0 = 0) before calculating the density. This latter thus will vary between those two marginal values. In a last step, these images are concatenated back together, and adjacent sub-images are averaged again with a Gaussian filter. The final result is a density map (Fig. 3A,B). Here an abstraction has to be made, because, as noted above, it is not cell density that is depicted, but a pixel density, which is related to -though stricto sensu not identical with- the former. For instance, in the core region, the cells are so dense that it is only possible to specify it as the maximum of density in this image. Therefore the core of the spheroid is always at the maximum value of 255. The gradient from the core towards the border quantifies the decrease of cell density. For a compact MCTS this decrease is steep (Fig. 3A), while for a spread-out core it is much softer (Fig. 3B). Figure 3C,D show the final result of the analysis as a surface plot that depicts the footprint of each MCTS. Even if cells migrate outside of the aperture, the increase in cell density in the border regions is reflected in the slope of the surface plot, thus compensating a possible loss of information. To obtain a measure of size, the density is integrated over the entire image.

This method evidences that it is not important to have the center of the MCTS of a sample at the exact same position on consecutive days, but only to be reasonably centered. As shown by Fig. 3C,D, this quantification method does not only measure the size of the core, but also its invasiveness. The invasiveness is reflected in the gradient away from the center. If a MCTS was only to proliferate but not to invade, the gradient would be relatively steep, since the core would keep its well-defined edges and just grow bigger as time progresses. On the other hand, if the MCTS were to only invade, this would be reflected in the slope becoming progressively lower while the core would stay constant, or even shrink if the assay is carried out long enough. Typically, both phenomena occur simultaneously, and the resulting progression shows an increase in the core size, as well as an opening up in outward direction from the center. Therefore, the result of our approach is not solely a measure of size, but a measure of overall aggressiveness. This way, a highly invasive core can be determined to be larger than a purely proliferative one, which is a desired result, since the cells belonging to the prior are more active.

In order to compare the sizes of different samples to each other, they need to be compensated for their differing initial sizes since it is technically not possible to create identical MCTSs. Therefore, the sizes at any time are expressed as relative to their own respective size at day 0. This has the advantageous side effect that the size units, which are proportional to the cell density but are not actual cell density, cancel out as well. With this approach, there is no need to define a cut off or a threshold for a minimum density value that would guarantee a reliable quantification. Indeed, the minimum density is accounted for by calculating relative sizes: any structure (e.g. impurities, extraneous cells) that is present from the beginning in the surrounding matrix and is recorded in the micrograph at day 0 will be canceled out of the result.

Finally, the image analysis does not only lead to a measure of the MCTS’s size, but it would be also useful for the automated quantification of fluorescent MCTS. Indeed, it provides a binarized abstraction of the original image, which can be used as a mask for filtering out unspecific fluorescent staining (data not shown).

### Evaluation of the approach: robustness and limitations

The robustness of the program has been extensively tested by using images of different qualities of the same MCTS, to demonstrate the response of the algorithm to various conditions, i.e. under- and over-exposing images, and the resulting camera noise and low contrast.

### Dark Images

**Figure 4:**
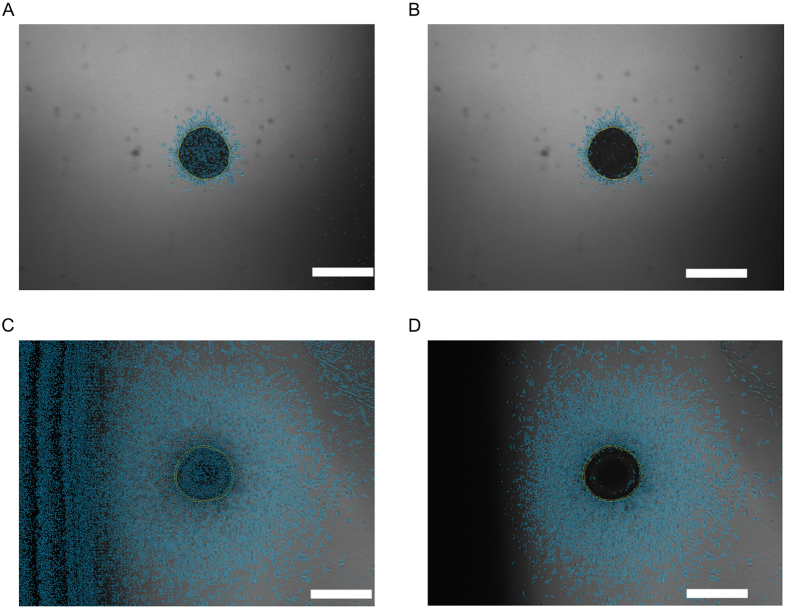
Algorithm response to camera noise and extreme borders. (**A,B**) Under-exposure introduces noise to the periphery. In this moderate case, it does not influence the final result, as indicated by the comparison of the image before (**A**) and after (**B**) background subtraction. (**C,D**) In case of extreme gradient, the periphery extraction may find false borders and noise due to low illumination in dark areas. In this case, a background subtraction prior to the periphery extraction must be performed. Comparison of the image before (**C**) and after (**D**) background subtraction indicates a substantial improvement. Any cells that may have been in the left border area are lost in the low illumination. We recommend to avoid such images. Scale bar = 500 μm.

Generally, under-exposing an image introduces camera noise. The periphery detection is based on an edge-detection algorithm, and therefore it is susceptible to camera noise. While the camera noise is acceptable in moderate cases, in extreme cases it may falsify the representation of the periphery. Furthermore, it is also influenced by strong brightness gradients, since they may appear as artificial edges, again falsifying the overall result in extreme cases. Should the result of an image be unsatisfactory, the analysis can be repeated with a background subtraction prior to periphery extraction (Fig. 4). A trade-off needs to be considered here. While the background subtraction is helpful in the presence of dark edges and extreme under-exposure, it causes the periphery in normally exposed images to be systemically too small. Therefore, it should be used only in exceptionally dark or strong gradient illumination conditions.

### Bright Images

**Figure 5:**
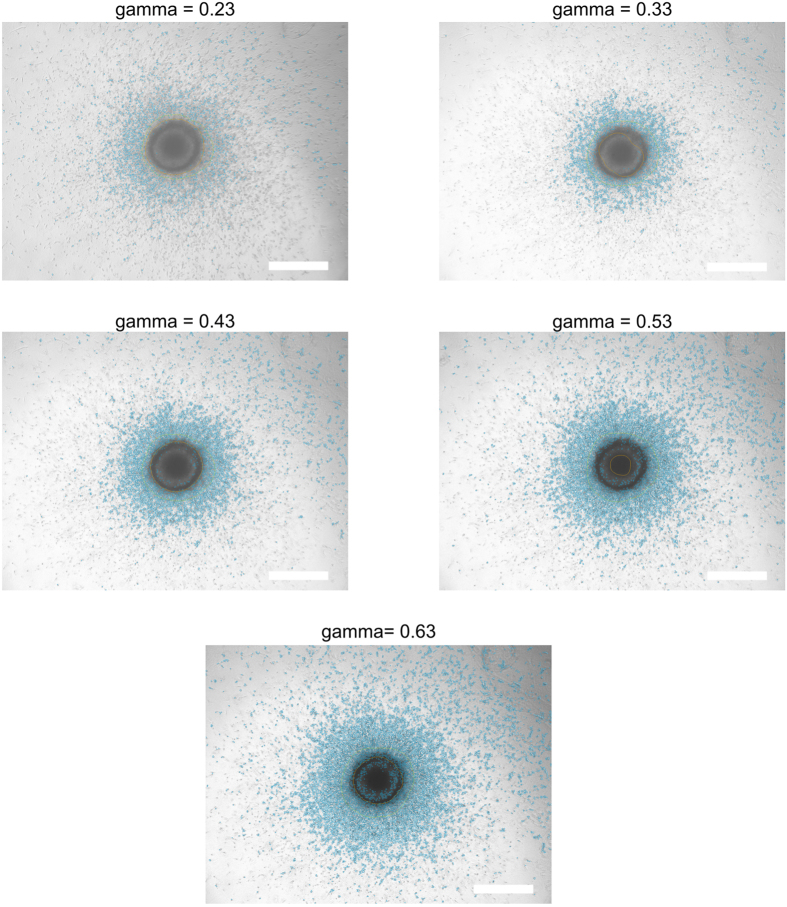
Impact of the contrast on the image processing. The same image was analyzed sequentially at different gamma levels in order to vary the contrast. A low contrast is typically observed in images that are taken at a high brightness. In this case, the images were taken at 100% brightness intensity. The segmentation improved with increasing gamma factor. Scale bar = 500 μm.

The contrast of an image affects the quality of the segmentation results. In order to demonstrate this effect, a series of images of the same MCTS taken at different gamma levels of the camera was analyzed with the brightness set at 100% (Fig. 5). As seen in the figure, the periphery extraction improves with an increasing gamma level. This shows a limitation of the algorithm for images with low contrast. This is an expected limitation, since a low gamma factor causes an effect in the image similar to that of a background subtraction. It may be useful to keep a low contrast for extremely uneven images but this will generally deteriorate he result. Therefore, it is important for the user to be aware of the quality of the produced images. Should they display a low contrast, it is advisable to increase the gamma factor for a correct evaluation through the software. Figure 5 can be utilized as a visual guideline for users.

### Reproducibility

**Figure 6:**
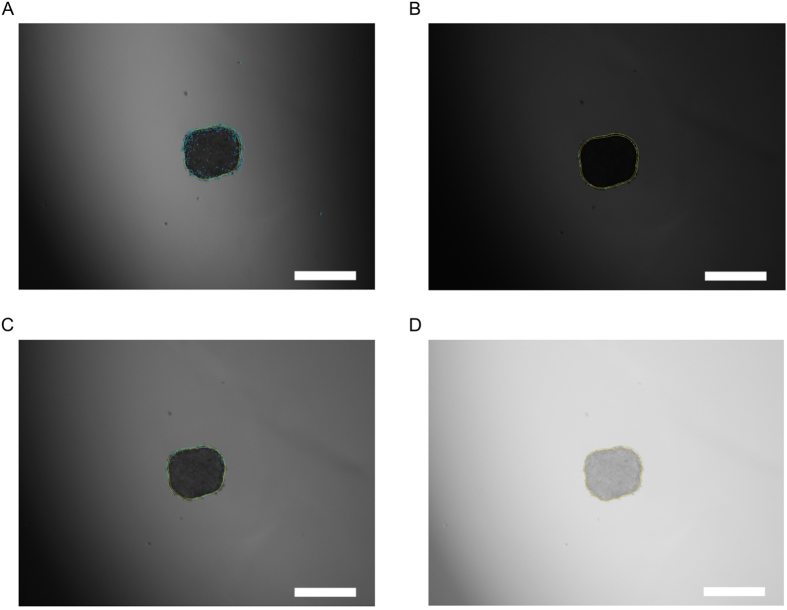
Example of the four different illumination conditions used for reproducibility testing. (**A**) Artificially introduced shadow. (**B**) Under-exposure. (**C**) Average condition. (**D**) Low contrast and high brightness. The average halo surface area of each condition was calculated along with standard deviation and these data are presented in Table 1. Scale bar = 500 μm.

The algorithms implemented in the program are deterministic without exception and therefore will always produce the exact same result for the same input image. Furthermore, it is stable to a wide range of illumination conditions. To demonstrate this, we estimated the error due to illumination by calculating the halo surface area of four MCTS under four different illumination conditions (Fig. 6). Images were acquired from four different samples under these varying conditions. The results are presented in Table 1. The average relative error of the analysis is approx. 2% of the calculated halo surface area, which is sufficiently low to demonstrate the reproducibility under varying illumination conditions. Usually, during the course of an assay, the illumination setup does not change as dramatically as depicted in Fig. 6. With less changes in illumination, the average relative error also reduces, so 2% relative error can be taken as an upper limit under extreme variations of illumination, i.e. worst-case estimation.

### False estimations of invasiveness

As part of this evaluation, we pondered upon determining the tendency of the algorithm for false-negative and false-positive estimations of invasiveness. However, we are not entirely sure how the terms false-negative and false-positive apply to our approach. Indeed, if there is no spheroid in an image, no spheroid will be found by the algorithm, hence no false positives are a possible occurrence. The same reasoning applies to false negatives. Expression of the quantified data as relative size to size at day 0 cancels out the background created by e.g. impurities in the collagen recorded on the micrograph. We could envisage that false-positive estimations result from the presence of cells in the collagen matrix that are dividing cells and not invading cells. However, it is very unlikely in the frame of a kinetics analysis, that cells present in the matrix would only be dividing cells and not dividing and invading (motile) cells. Analysis with the software obviously does not give any information on which biological process is at work. For that purpose, one has to analyze cells of the spheroids and cells in the collagen and measure directly their proliferative status. Finally, false estimations might result from failure of the segmentation. According to our experience, this is due to poor image quality, or by accidentally selecting the wrong micrographs. In summary, given the current state of development of the software and its robustness, we are confident that the risk of false estimations of invasiveness is very low and does not represent a limitation to the use of the software.

### Proof of concept

The program has been used for the analysis of numerous invasion assays and has been under constant development. As proof-of-concept, we provide a selection of invasion assays and their corresponding results.

### Invasion Profiles

**Figure 7:**
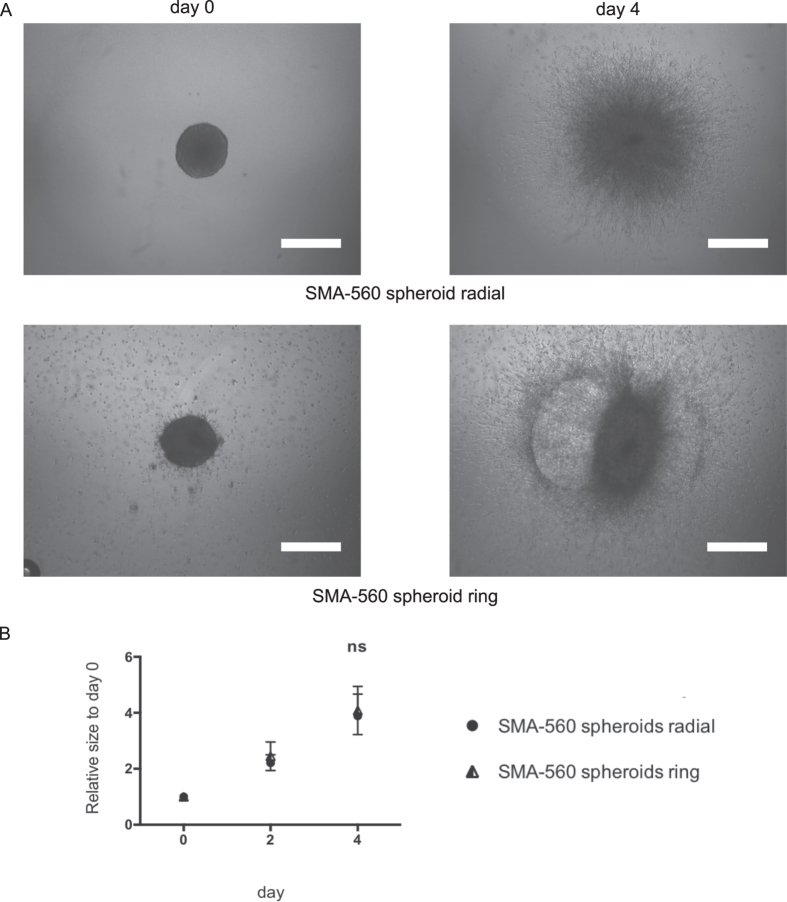
Invasion of murine astrocytoma SMA-560 MCTS. (**A**) Representative images of radial and ring invasion profiles of SMA-560 MCTS at day 0 and day 4 respectively. All images were acquired with an inverted light microscope at 4x magnification. Scale bar: 500 μm. (**B**) Quantification of invasiveness of SMA-560 MCTS. Data is expressed as mean ± standard deviation of the relative size of the MCTS at the indicated time. Results are from three independent experiments. ns: not significant.

Not all MCTSs invade homogenously in all directions. The invasion pattern depends on the competition between cell-cell and cell-matrix interactions^15^. Figure 7 clearly shows two different invasion profiles of MCTSs, generated with the same murine glioma cell line (SMA-560), embedded in a collagen matrix and run under the same conditions. As seen in the representative images of Fig. 7A, the MCTS have comparable starting conditions at day 0; however, the upper MCTS displays a homogeneous radial invasion, whereas the lower MCTS generates ring-like structures during invasion. Regardless of the different profiles, the software is still able to quantify the invasiveness correctly in each case (Fig. 7B). We observe that there is no statistical difference in the invasiveness of the two profiles, which corresponds to the images in Fig. 7A. This demonstrates the strength of the software to be independent from MCTS invasion profiles, thanks to the approach of estimating binarization density.

### Invasion assays with different glioma models

**Figure 8:**
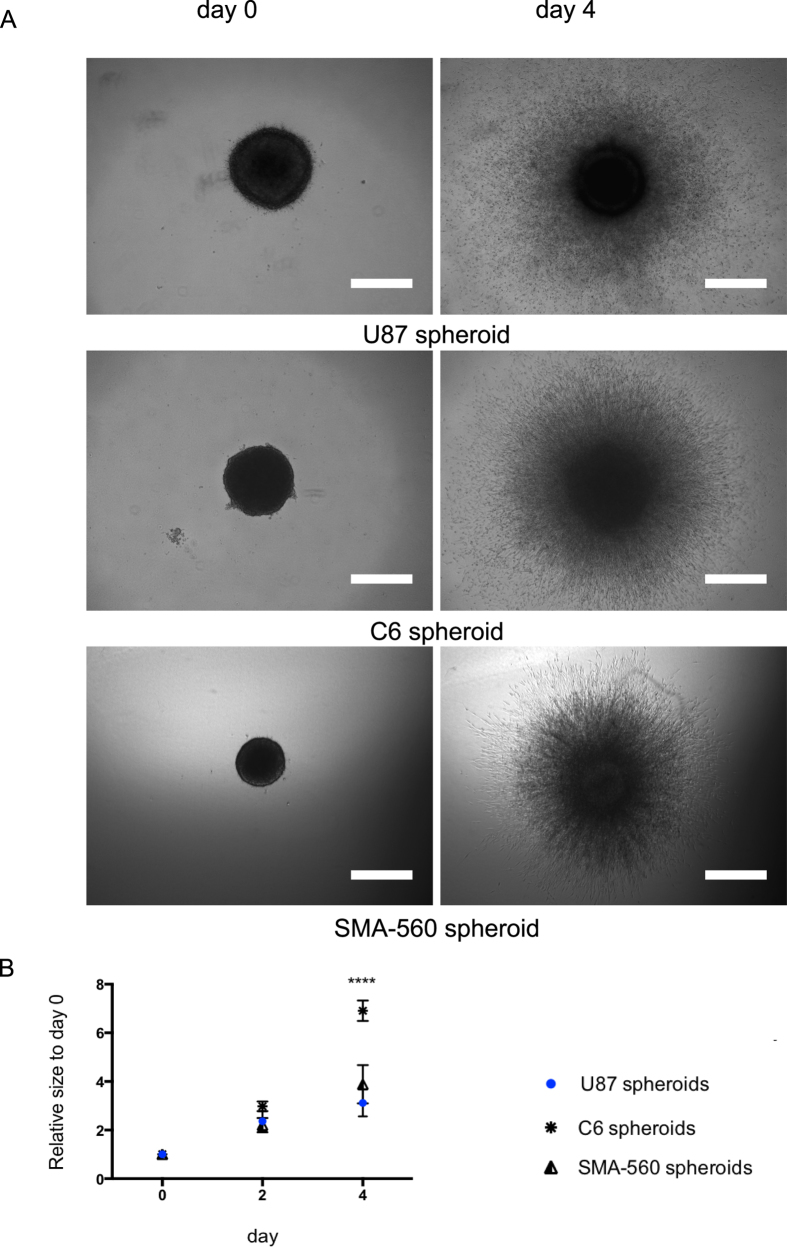
Invasion of different models of glioma MCTS. (**A**) Representative images of three different glioma cell line MCTS (U87MG, C6, SMA-560) at day 0 and day 4. All images were acquired with an inverted light microscope at 4x magnification. Scale bar: 500 μm. (**B**) Quantification of invasiveness of the MCTS of the three cell lines. Data is expressed as mean ± standard deviation of the relative size of the MCTS at the indicated time. Results are from three independent experiments. ****p < 0.0001.

When studying glioma signaling or drug treatment response in 3-D models, it is important to characterize the basic invasion behavior of different cell lines in which these signaling or drug responses are being analyzed for validation purpose. Figure 8 depicts the quantification (Fig. 8B) of the results of invasion assays carried out for MCTSs generated from three different glioma cell lines (U87MG, C6, SMA-560) and embedded in the collagen matrix (representative images in Fig. 8A). As observed in Fig. 8A, all MCTSs show a comparable invasive area at day 4, suggesting a similar invasiveness. However, they do have distinct initial sizes, which casts doubt on the previous suggestion. Indeed quantification of the relative size for the cell lines at different time points indicates different invasion profiles (Fig. 8B). At time point day 2, we observe no statistically significant differences in the relative size, i.e. in invasiveness between the three conditions. At time point day 4, the situation changes: the relative sizes of the U87MG and SMA-560 MCTSs are still similar (no statistical difference), whereas that of C6 MCTSs is statistically significantly higher, indicating a higher invasiveness compared to U87MG and SMA-560 MCTSs. The relative sizes we can measure with the help of the software are the results of two biological functions of the cells: proliferation and invasion, which cannot be discriminated by the software. The visual comparison of the dense core regions at day 0 and day 4 (Fig. 8A) however is helpful in that matter. It clearly shows that the size of the initial dense core region of the U87MG MCTS diminishes with time, concomitantly to the outward migration of cells. This suggests that U87MG cells do migrate but do not proliferate to an extent that maintains the initial size of the core region. The initial dense core region of the C6 MCTS on the contrary increases with time concomitantly to the outward migration of cells. This suggests that the C6 MCTS are more proliferative (increase of the initial core) than the U87MG MCTS; moreover, they most likely are more invasive than U87MG MCTS as well, given their smaller initial core region and an area of invasion similar to that of U87MG MCTS observed in the micrograph at day 4. This example demonstrates the advantage of the relative size measurement.

### Effect of microglia immunomodulation on glioma biology

**Figure 9:**
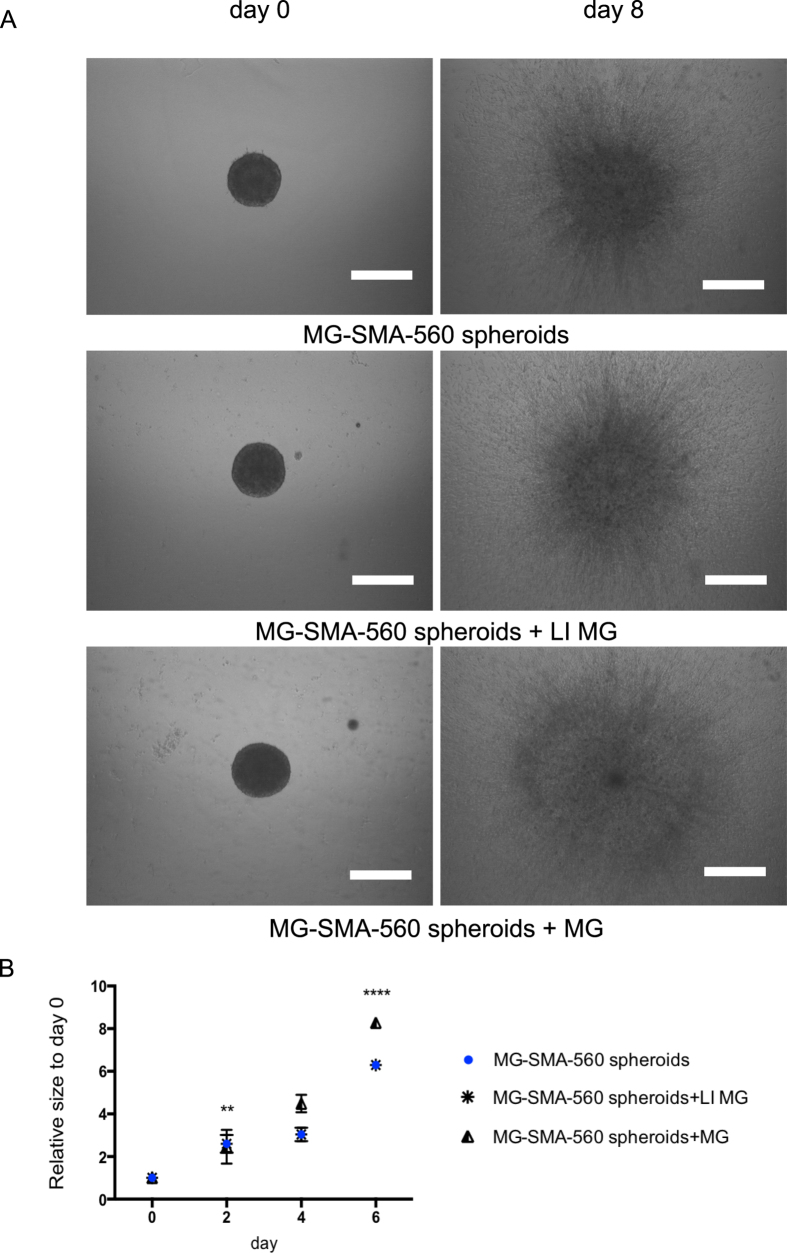
Invasion of mixed microglia (MG)-SMA-560 MCTS in the presence or absence of M1-like microglia (LI MG) or untreated microglia. (**A**) Representative images of the invasion of MG-SMA-560 MCTS in collagen in absence or presence of MG at day 0 and day 8. All images were acquired with an inverted light microscope at 4x magnification. Scale bar: 500 μm. (**B**) Quantification of invasiveness of the MG-SMA-560 MCTS. Data is expressed as mean ± standard deviation of the relative size of the MCTS of the indicated time. Results are from three independent experiments. **p < 0.01, ****p < 0.0001.

Tumor-associated inflammation is triggered by the infiltration of glioma-associated microglia/macrophages (GAMs) and the production of cytokines and chemokines. The tumor environment gradually inhibits the immune response of GAMs (M1-like polarization) against the tumor cells through various soluble mediators and mechanisms, and the glioblastoma microenvironment remains immunosuppressive and polarizes GAMs towards an M2-like and tumor supportive status^16^. As part of a project that aims at analyzing interactions between microglia and tumor cells, we set up the following approach, using primary murine microglia and the murine astrocytoma cell line SMA-560. Murine primary microglia were left untreated (MG) or treated with lipopolysaccharide (LPS) and interferon-gamma (LI MG) for 48 h and thereafter included in the collagen matrix containing mixed murine microglia-SMA-560 cells spheroids. Figure 9A shows representative micrographs at day 0 and day 8 of the invasion assay. Quantification of the images and the resulting kinetics analysis are shown in Fig. 9B. At day 2, there are no significant differences in the relative sizes of the various conditions. Beginning from day 4, differences in the relative sizes that are statistically significant begin to appear and persist until the last time point. These differences indicate that, as expected^11^, the untreated MG increased the invasiveness of the mixed spheroids when compared to the control condition (absence of microglia in the collagen matrix) whereas the treated MG, despite exhibiting a cytokine M1-like profile (data not shown) did not affect invasiveness, suggesting an immunosuppressive activity of the tumor cells. This assay is another demonstration of the robustness of the software. Indeed the presence of an additional visible cell type into the ECM might have increased the binarization density. However, due to the relative units, the offset that comes with the cells is canceled out of the results.

### Effect of combined treatment of TMZ and SKI on GBM MCTS

**Figure 10:**
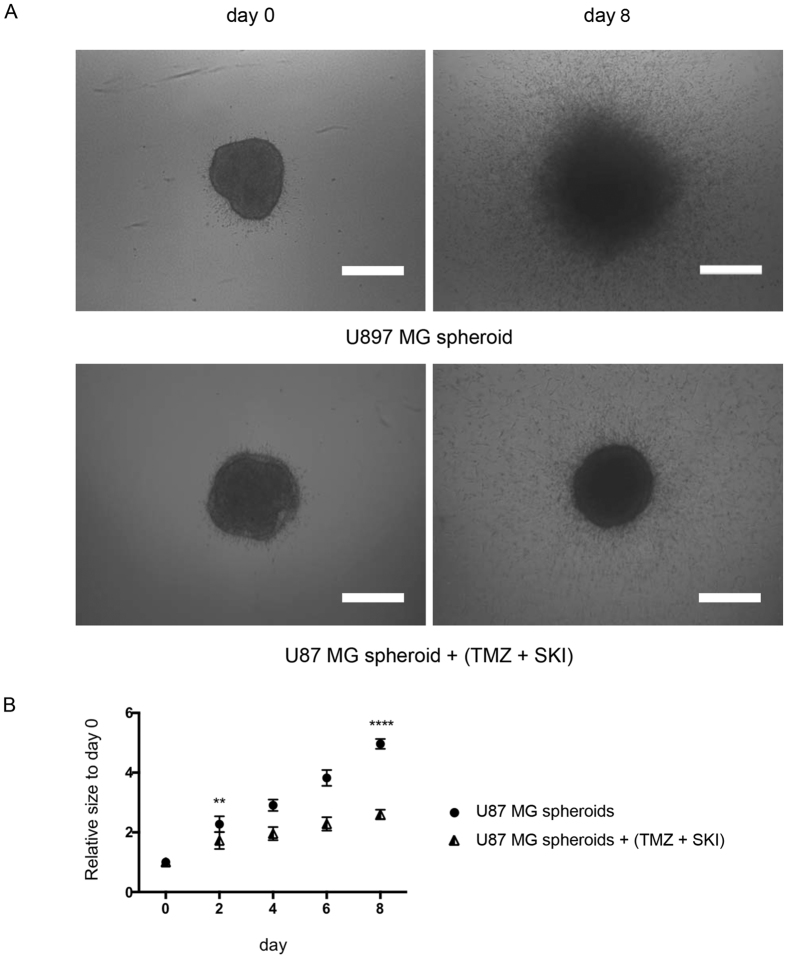
Invasion of tumor (U87MG) MCTS in the presence or absence of (25 μM TMZ + 10 μM SKI) treatment. (**A**) Representative images of U87MG MCTS treated and untreated at day 0 and day 8. All images were acquired with an inverted light microscope at 4x magnification. Scale bar: 500 μm. (**B**) Quantification of invasiveness of U87MG MCTS. Data is expressed as mean ± standard deviation of the relative size of the MCTS of the indicated time. Results are from three independent experiments. **p < 0.01; ****p < 0.0001.

We previously showed that combination of a sphingosine kinase inhibitor (SKI) with temozolomide (TMZ) increases human glioblastoma cell death when compared with singular treatment regimen^17^. In the frame of these proof-of-concept experiments, we investigated the effects of this combination on the invasiveness of U87MG MCTS embedded in the collagen matrix (Fig. 10). Representative images in Fig. 10A clearly show a visible effect of the combined treatment on the invasiveness of the MCTS. Quantification of the images with our software (Fig. 10B) indicates statistically significant differences in the relative sizes of the conditions as early as day 2. These differences increase with time until the last tested time point. These results provide another validation of our approach to spheroid image analysis.

### Summary

The automated approach was developed with two main related scopes in mind: improvement of efficiency and accuracy of the analysis on the one side, and reduction of the human influence on the result on the other side. We provide evidence in this report for both aims. The approach is robust to various imperfections of images as well as to cell-related issues (compaction, invasion profiles). It reduces human influence and therefore human error and bias in the results since human intervention is limited to the photography steps and loading of images in the program. Compared to other automated approaches, the algorithm we have designed has another, cost-related and practical advantage because it does not require a specific equipment and can process images taken by any camera connected to a microscope. Thus, the program can be used by any experimenter. The originality and the strength of the algorithm consists is stepping away from cell counting and defining an invasive front, which is not always possible in a two-dimensional representation of a three-dimensional object, and moving towards the quantification of the density of this object. Here, the result is not a density given in cells per area, but it is an inferred density, based on the segmentation of the object. To the best of our knowledge, we present the first quantification approach through binarization density for the analysis of invasion assays performed with MCTSs embedded in an ECM. Implementation of this approach shall contribute to a larger use of these three-dimensional models as it greatly facilitates the generation of accurate and robust data.

## Methods

### Ethics Statement

Handling and sacrifice of neonates (P0) mice were performed in accordance with institutional guidelines of the German Cancer Research Center (DKFZ, Heidelberg) and approved by the German Federal State of Baden-Württemberg, in accordance with the European Community Council Directive of November 24, 1986 (86_609_EEC).

### Cells and cell culture

The human glioblastoma cell line U87MG was purchased from the American Type Culture Collection (ATCC). Rat C6 glioma cell line was kindly provided by Prof. Dr. Bernd Kaina (Institute of Toxicology, University Medical Center, Mainz, Germany). A malignant spontaneous murine astrocytoma (SMA-560) was used as a murine glioblastoma model^18^,^19^. Murine primary microglia (MG) were isolated from the brain of newborn VM/Dk mice that were bread at the German Cancer Research Center (DKFZ, Heidelberg, Germany) animal facility as described^20^. Cells were cultured in high-glucose Dulbecco’s Modified Eagle’s Medium (DMEM) and supplemented with 10% heat-inactivated fetal calf serum (FCS), 2 mM L-glutamine and 50 μg/ml gentamicin, all from Sigma-Aldrich. This medium is referred to as complete DMEM (cDMEM). The FCS concentration was reduced to 5% in medium used for experiments (c-DMEM-5). Cells were maintained in standard culture conditions (37 °C in humidified air with 5% CO_2_).

### Generation of multicellular spheroids

#### Manual hanging drop technique

##### Tumor cell spheroids

U87MG and SMA-560 cells expanded in cell-culture flask were detached with 0.25% trypsin/EDTA (Invitrogen) and re-suspended with cDMEM at the final concentration of 10^6^ cells/ml. 25 μl of this cell suspension (i.e. 25000 cells) were deposited in a drop-like form on the lid of a 10 cm Petri dish which was flipped back to the dish containing 8 ml of phosphate-buffered saline (PBS). Drops were incubated at standard culture conditions for 96 h.

##### Mixed cells spheroids

Murine primary MG and SMA-560 cells were detached by trypsinisation and mixed. A cell suspension of 10^6^ cells/ml (0.5*10^6^ cell/ml MG and 0.5*10^6^ cell/ml SMA-560 cells) in cDMEM was prepared. Drops of 25 μl of this cell suspension (i.e. 25000 cells) were plated on the lid of a 10 cm Petri dish, which was flipped back to the dish (containing 8 ml of PBS). Drops were incubated at standard culture conditions for 96 h and thereafter transferred to agar-coated tissue cultured dishes in 7ml of cDMEM for 48 h before embedding in collagen type I. Generation of MG-SMA-560 spheroids indeed require this additional step to facilitate the formation of spheroids of uniform morphology and size.

#### GravityPLUS hanging drop system

U87MG cells prepared as described above were resuspended in cDMEM at the final concentration of 25000 cells in 40 μl. Cells were distributed (40 μl/well) in the GravityPLUS 96-well plate according to the manufacturer’s instructions (InSphero). Plates were incubated at standard culture conditions for 72 h.

#### Spheroid Microplates

C6 rat glioma cells were resupended in cDMEM at the final concentration of 12500 cells in 200 μl. Cells (200 μl per well) were distributed in flat 96-well low attachment surface plates (Corning). Plates were incubated at standard culture conditions for 96 h U87MG cells prepared as described above were resuspended in cDMEM at the final concentration of 25000 cells in 200 μl. Cells (200 μl per well) were distributed in flat 96-well low attachment surface plates (Corning). Plates were incubated at standard culture conditions for 96 h.

### 3-D invasion assay

After 72 or 96 h of culture, U87MG, C6, SMA-560 cell spheroids were implanted in the center of each well of a 24-well plate coated with a 2.2 mg/ml collagen mixture (one spheroid per well in 400 μl of collagen mixture per well). For each of the three independent experiments, 10 spheroids generated by each cell line were randomly chosen for embedding. The collagen mixture was prepared by mixing 2 ml of PureCol bovine collagen type I solution (3 mg/ml; Advanced BioMatrix) with 250 μl of 10X minimal essential medium (MEM) (Sigma-Aldrich) and 500 μl of sodium hydroxide 0.1 M. After cell spheroid embedding, the plate was incubated for 20 min at standard culture conditions to solidify the gels. Thereafter 400 μl of cDMEM was overlaid on the collagen matrix in each well. The complete system was incubated for a total of 8 days.

### Sphingosine kinase inhibitor (SKI) and Temozolomide (TMZ) treatment of tumor cell spheroids

The effect of drug treatment was assessed using SKI (Sigma; 33 mM in DMSO, stock solution) and TMZ (Sigma-Aldrich, 160 mM in DMSO, stock solution). The stock solutions of SKI and TMZ were diluted in MEM 10X at the final concentration of 10 μM of SKI and 25 μM TMZ. The MEM 10X containing the drugs was mixed with the other components of the collagen mixture and distributed in 24-well plates for spheroids embedding as described in C. Spheroids without treatment are considered the control condition for this experimental set-up; these spheroids were embedded in collagen containing a volume of dimethylsulfoxide (DMSO) equal to that of treatment. This volume (0.1% v/v) had no effect (data not shown).

### M1-like *ex vivo* activation of primary murine microglia

Primary murine microglia were treated with a combination of lipopolysaccharide (0.1 μg/ml final concentration) and interferon γ (33 ng/ml final concentration) in cDMEM with 5% FCS for 48 h. Treated microglia (MG LI) and untreated microglia (MG) were trypsinised, resuspended in MEM 10X and mixed with components of the collagen mixture as described in C. The collagen mixture containing microglia cells was distributed in 24-well plates (400 μl/0.25*10^6^ microglia per well) for embedding of MG-SMA-560 spheroids (prepared as described in section B) as described in section C. Mixed spheroids embedded in collagen lacking microglia cells were used as controls.

### Quantification of spheroid size and invaded area

After the spheroids were embedded, cell invasion out of the spheroid was monitored by digital photography using a Leica DM IL LED Fluo inverted light microscope (Leica DFC450C camera) at room temperature (RT), with the Leica Application Suite (LAS V4.4). Images were acquired every 24 h (day 0 = time of embedding in collagen; picture taken 3 h after embedding) using a 4x/0.10 objective. Image processing and quantification of spheroids and of invasion areas was performed using the in-house software described in this manuscript. Invasion of the spheroids are normalized for each day to the invasion of the spheroids measured at day 0. These normalized data are reported as relative size to day zero. Relative size of day 0 thus equals 1.

Quantification was performed with a maximum of 30 samples (10 spheroids/experiment, three independent experiments). We excluded from the quantification images of spheroids that displayed post-embedding (thus unrelated to the generation method) abnormalities in matrix morphology (loss of structure). In the current stage of the software, the processing takes approx. 30 seconds per image on a standard computer. This time can be further optimized, if needed, by running several images in parallel.

### Statistical Analysis

All statistical analyses were performed using Prism (GraphPad). All data are expressed as mean ± standard deviation (SD) of at least 3 independent experiments. Statistical significance of data obtained from at least 3 independent experiments was determined using two-way ANOVA analysis of variance (ANOVA) combined with Bonferroni analysis with P < 0.05 being considered statistically significant.

The data we utilized for these statistical analysis were all first subjected to a series of quality tests we describe in ref. 21 and that are not shown here. We namely determined the factor [ratio of reproducibly invading spheroids per experiment] that we named linearity-over-yield and applied it to correct the data generated.

## Additional Information

**How to cite this article**: Cisneros Castillo L.R. *et al*. A Novel Computer-Assisted Approach to evaluate Multicellular Tumor Spheroid Invasion Assay. *Sci. Rep.* **6**, 35099; doi: 10.1038/srep35099 (2016).

## Supplementary Material

Supplementary Information

## Figures and Tables

**Table 1:** Analysis of reproducibility under varying illumination conditions.

	Average surface area [μm^2^]	Standard deviation [μm^2^]	Relative standard deviation
Sample A	150032	2208	1.47%
Sample B	156045	3411	2.19%
Sample C	164656	3230	1.96%
Sample D	151778	2885	1.90%

Average relative error 1.88%.

Four different samples were imaged under four different illumination conditions (Fig. 6). Their average halo surface area was calculated along with their standard deviation, which is taken as a measure of illumination-induced variance. The standard deviation is given in percent relative to the average size. The overall relative error is the average of the individual relative deviations. As seen in the table, the average is at around 2% of the estimated value, proving the robustness under different illumination conditions.
