# Supplementary material for: A Novel Computer-Assisted Approach to evaluate Multicellular Tumor Spheroid Invasion Assay
Source: Sci Rep. 2016 Oct 12;6:35099. doi: 10.1038/srep35099 (PMC5059692; doi:10.1038/srep35099)
Supplement: Supplementary Information [file srep35099-s1.pdf]

Supplementary Material

A Novel Computer-Assisted Approach to evaluate Multicellular Tumor Spheroid Invasion Assay

Liliana R. Cisneros Castillo[1], Andrei-Dumitru Oancea[2], Christian Stüllein[2], and Anne Régnier-Vigouroux[1],*

[1]Institute of Zoology, Johannes Gutenberg University of Mainz, Mainz, Germany

[2]CLADIAC GmbH, Heidelberg, Germany

*Corresponding Author:

Anne Régnier-Vigouroux

Johannes Gutenberg University of Mainz,

Johann-Joachim-Becher-Weg 15

55128 Mainz, Germany

Tel.: 0049-6131-39 23 949

No fax number

E-mail address: vigouroux@uni-mainz.de

Condensed title:

Analysis of MCTS Invasion Assays

Figure S1

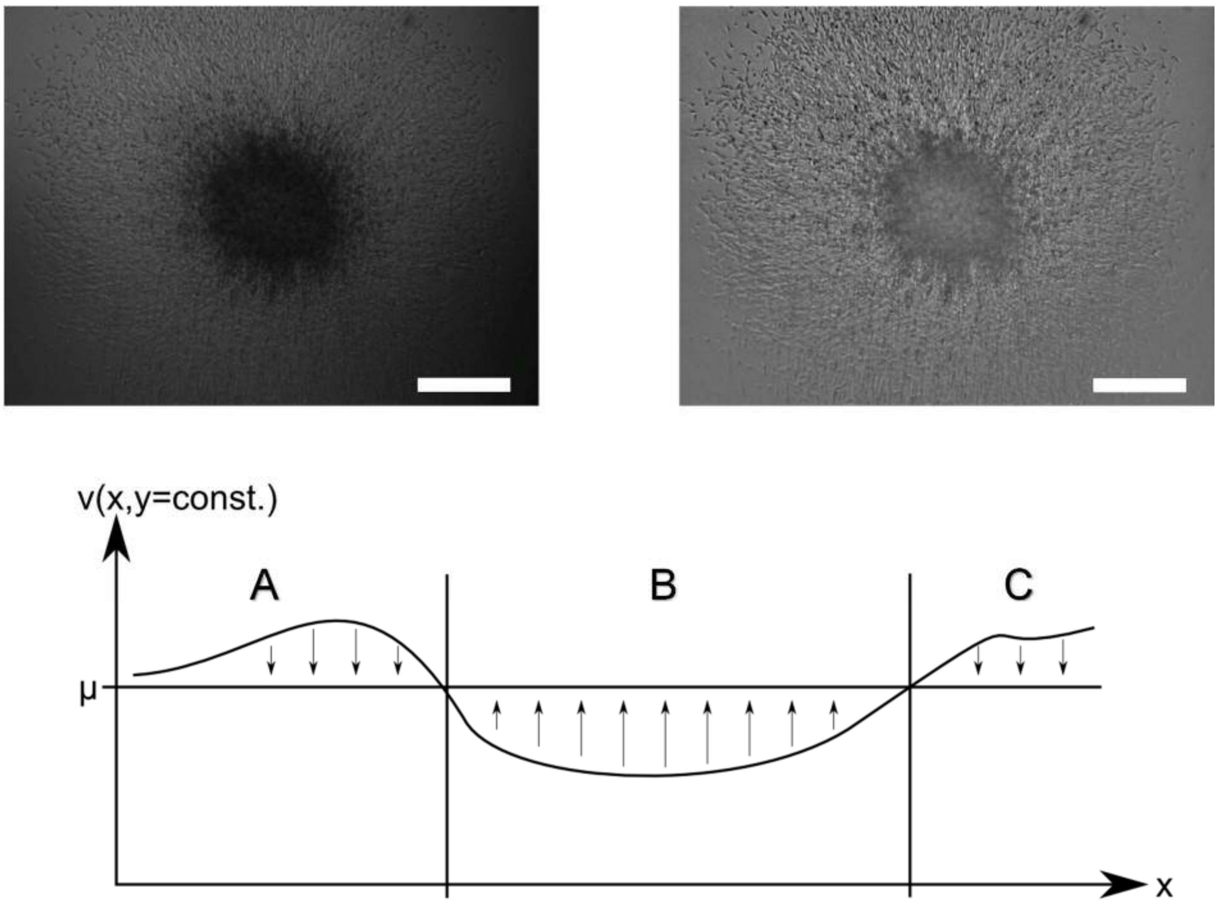

Figure S1: Visual explanation of the background subtraction. Top: image of a spheroid before (left) and after (right) background subtraction. Bottom: the uneven black curve depicts the intensity v along x direction on a constant y coordinate. μ is the previously determined overall average value of the image. The subtraction algorithm loops through the image pixel-wise and subtracts the excess value if the pixel is above average (seen on the border sections A and C), or adds the value needed to reach average (seen in the center section B). The result is an image, in which smooth illumination gradients are adjusted while maintaining the perturbations that occur on a small scale. Scale bar = 500 μm.
